# Effects of 2,4,6-Trichlorophenol on *Clarias batrachus*: a biomarkers approach

**DOI:** 10.1007/s11356-022-19213-y

**Published:** 2022-02-17

**Authors:** Dip Mukherjee, Nuno G. C. Ferreira, Nimai Chandra Saha

**Affiliations:** 1 Department of Zoology, S.B.S. Government College, Hili, Mera Aptair, Balurghat, Dakshin Dinajpur-733126 West Bengal India; 2 School of Biosciences, Cardiff University, Cardiff, CF10 3AX UK; 3 CIIMAR, Terminal de Cruzeiros de Leixões, Av. General Norton de Matos S/N, 4450-208 Matosinhos, Portugal; 4 The University of Burdwan, Fishery and Ecotoxicology Research Laboratory, Vice Chancellor’s Research Group, Department of Zoology, University of Burdwan, Purba Barddhaman, West Bengal India

**Keywords:** Chlorophenols, Physiological parameters, Reproduction impact, IBR

## Abstract

2,4,6-Trichlorophenol (2,4,6-TCP) is a common waste among the resulting chlorophenols generated in the production of common products classified as an extremely toxic, mutagenic, carcinogenic and highly persistent xenobiotic in the environment. To evaluate the impact of 2,4,6-TCP in aquatic systems, the catfish species *Clarias batrachus* has been selected to test its toxicity due to its high market value and consumption in India. Here is presented the impact of this compound on different physiological parameters of fish: haematological parameters (haemoglobin, total erythrocyte count, total leucocyte count and mean corpuscular haemoglobin), biochemical parameters (total serum protein and total serum glucose), growth and reproductive parameters (condition factor, hepatosomatic index, maturity index, specific growth rate, growth hormone, 17β-estradiol and testosterone), exposed to two concentrations of 2,4,6-TCP (0.5 mg/L and 1 mg/L — 1/10th and 1/20th of the LC_50_) for a period of 15, 30 and 45 days. The results showed that *C. batrachus* even when exposed to the lower concentration (0.5 mg/L) for the shortest time (15 days) negatively impacted the organism in all the assessed parameters. This was highlighted by the Integrated Biomarker Response index (IBR), showing worse scores for the treatments (up to 20 × worse than the control). This work highlights the importance of continued research on the impact of 2,4,6-TCP, on an important commercial, supported by the high environmental persistence of this compound that can reach the same range of tested concentrations.

**Supplementary Information:**

The online version contains supplementary material available at 10.1007/s11356-022-19213-y.

## Introduction

The increased discharge of various chlorophenolic compounds is a significant cause of concern for the environment (U.S.EPA 2017). One example of products included in this group is 2,4,6-trichlorophenol (2,4,6-TCP). This chlorinated phenol is widely used as an antiseptic, glue, leather and wood preservative, antimildew agent, water chlorinator, organic solvent and the synthesis of various agricultural chemicals (Olaniran and Igbinosa 2011). 2,4,6-TCP has been classified as an extremely toxic, mutagenic and carcinogenic compound due to the C–CL bond position relative to the − OH group. This makes it recalcitrant to biodegradation and leads to its interference in mitochondrial oxidative phosphorylation through the inhibition of cytochrome P450-dependent mixed-function oxidases (NCBI 2021). The recalcitrant nature of 2,4,6-TCP results in high toxicity for a considerable period (Benbachir et al. 2017). This chemical enters the environment through several routes like industrial waste, insecticides or by degradation of complex chlorinated hydrocarbons. It can bioaccumulate, for example, in fish, as observed in the studies of Igbinosa et al. (2013) and Muir and Servos (2020). In the environment, 2,4,6-TCP has been quantified in groundwater (91.3 ppb), in surface waters in Canada (up to 30 ppb), in sediments of Canadian streams (up to 10 mg/kg) and in drinking waters of the USA, Finland and Canada (0.014–0.7 ppb, NTP-DHHS 2016). Its occurrence has also been extensively recorded, in China, and 54.4% of the 600 sampled sites in seven major watersheds and three drainage areas showed contamination by 2,4,6-TCP (Jin et al. 2012). Thus, this chemical poses a significant risk to various target and non-target organisms linked by the food chain in the aquatic ecosystem (Wang et al. 2021).

In chronic toxicity studies on fish, chlorophenols have been reported to cause a wide range of negative impacts such as oxidative stress, carcinogenesis and reproductive toxicity (Fang et al. 2014; Ge et al. 2017); alterations in plasma steroids, liver size, sexual maturity, fish growth and survival (Sepúlveda et al. 2003; Van Der Kraak et al. 1992) and various condition indices (e.g. condition factor, hepatosomatic and gonadosomatic index, Ussery et al. 2021). Since biomarkers can act as indicators of environmental pollutants, their use to evaluate the toxicity of 2,4,6-TCP will provide key information (Jemec et al. 2016). The primary signal indicative of potential damage inflicted by xenobiotics to various cellular and subcellular tissues/organs of an organism are elicited by multiple sets of biomarkers (Ferreira et al. 2015b). In this sense, the use of the Integrated Biomarker Response (IBR), a comprehensive and popular tool that aids in the analysis between levels of pollution and multiple biomarkers, biomonitoring of stress and graphical interpretation of the various biological effects of xenobiotics will greatly increase the data analysis (Beliaeff and Burgeot 2002, Devin et al. 2014; Zheng et al. 2013). Thus, the combined use of biomarkers in IBR can be a useful tool in ecotoxicological assays, providing data that cannot be so easily observed.

Although pentachlorophenols have been widely studied, scarce information is available on the chronic toxic effects of 2,4,6-TCP on fish. To help fill this gap, the air breathing catfish *Clarias batrachus* was chosen as the test species for this study. *C. batrachus* is a species that easily acclimate to laboratory conditions, is easy to maintain and is used in ecotoxicological tests (Mohindra et al. 2012). In addition, it is commonly distributed in freshwater ecosystems throughout India. This species serves as a food and medicinal source with high market value to local populations, thus also presenting itself as a possible route for the ingestion of chemicals (Agarwal et al. 2017; Sahoo et al. 2015).

The present study aims to determine the following: (1) the chronic toxic effects of 2,4,6-TCP on haematological parameters (haemoglobin, total erythrocyte count, total leucocyte count and mean corpuscular haemoglobin), biochemical parameters (total serum protein and total serum glucose), growth and reproductive parameters (condition factor, hepatosomatic index, maturity index, specific growth rate, growth hormone, 17β-estradiol and testosterone) of air breathing catfish, *Clarias batrachus*; and (2) the use of IBR as a tool to determine the deleterious effects of this chemical not always evident.

## Materials and methods

### Experimental animal

Adult specimens of the air breathing catfish, *Clarias batrachus* (*n* = 120), were collected from a local fish farm at Naihati (District North 24 Parganas, West Bengal) and transported to the Aquatic Toxicology laboratory (Barasat Government College, West Bengal). Fish (weight 106.3 ± 2.2 g; length 17.8 ± 0.8 cm) were acclimated to laboratory conditions for 2 weeks in flow-through outdoor tanks (6000 L capacity) with dechlorinated water (pH 7.4–7.9) under natural photoperiod (12:12 h light–dark) and ambient temperature. A constant supply of oxygen to the tanks was ensured through air pumps (Aquaspeed AP-446). They were fed ad libitum with dried organic fish mini sinking pellet (CPF India Pvt. Ltd.). After acclimation, fish were divided into two sets in separate aquaria of 6000 L (males and females). The water quality was maintained by partial renewal (25–30%) every 2 days.

### Experimental design

#### Experimental design: chronic exposure

The chronic toxicity tests were carried out using 2,4,6-Trichlorophenol (CAS No. 88–06-02, Sigma Aldrich, St. Louis, USA, 98% purity). The stock solution was prepared in dechlorinated water with 1% dimethyl sulfoxide that was then added to the 1000-L tanks (please see description below), making the final solvent concentration neglectable (0.0004%). As a result, the inclusion of a solvent treatment was not required. A control and two treatments (0.5 mg/L and 1.0 mg/L), corresponding to 1/20th (0.5 mg/L) and 1/10th (1 mg/L) of the 96-h LC_50_ determined for *C. batrachus* exposed to 2,4,6-TCP as reported in a previous study for 45 days (9.95 mg/L, Mukherjee 2015) were used in this study. The duration of the experiment was also of 45 days, with sampling being done after 15, 30 and 45 days of exposure. 2,4,6-TCP was measured using Gas Chromatography/Mass Spectrometry (GC–MS — EPA 3510 and EPA 8270). Since differences between nominal and measured values were lower than 10%, nominal concentrations were used throughout the manuscript. Adult fish (four male and four female — each corresponding to one replicate) were added to the experimental tanks (1000 L capacity) for the measurement of the haematological, biochemical, growth and reproductive parameters in a randomised design (Gomez and Gomez 1984). The medium in all the replicates was entirely renewed every 5 days with a fresh-made solution of 2,4,6-TCP to maintain water quality and concentration (physico-chemical parameters of water are summarised in Table S1 Supplementary Data). The fish were fed three times a day until they were visually satiated.

### Collection of blood, serum and assay of haematological profiles and indices

The fish were first anaesthetised using clove oil (60 µl/L water) to avoid stress. Blood and serum were collected on day 15th, 30th and at the end of the exposure period (45th day). Blood was taken by puncturing the fish’s caudal vessels using a 4-ml 23G Dispovan syringe previously rinsed with 3% EDTA solution. The blood was immediately transferred to vacutainer EDTA coated tubes (Becton Dickinson, USA) and mixed well to avoid blood clotting. Another fraction of blood was collected without EDTA and spun in a cooling centrifuge at 3000 rpm for 20 min at 4 °C. The yellow coloured serum was collected with a micropipette, transferred to microtubes and stored at − 20 °C for further analysis of total serum protein (TSP), total serum glucose (TSG), testosterone (T), 17β-estradiol (E_2_) and growth hormone (GH) that took place no longer than 2 days.

The haemoglobin level in blood was estimated by Sahli’s method using 0.1 N HCl (Briggs and Bain 2017). The total erythrocyte count (TEC, 10^6^/mm^3^) and total leucocyte count (TLC, 10^3^/mm^3^) were estimated following standard procedures (Briggs and Bain 2017). The TEC (10^6^/mm^3^), TLC (10^3^/mm^3^) and Mean Corpuscular Haemoglobin (MCH, pg) were calculated using the formulas described as follows:

TEC (10^6^/mm^3^) = [Total number of cells counted in Neubauer Haemocytometer × dilution factor (200)] / [1/5 × volume factor (0.1)].

TLC (10^3^/mm^3^) = [Total number of cells counted in Neubauer Haemocytometer × dilution factor (50)] / [4 × volume factor (0.1)].

MCH (pg) = [Haemoglobin (g/dL) × 10] / TEC (10^6^/mm^3^).

### Analyses on growth and reproductive endpoints

Condition Factor (K), Hepatosomatic Index (HSI), Maturity Index (MI) and Specific Growth Rate (SGR) were measured at 15 days intervals for the entire exposure period of 45 days. For K, HSI and MI, at the end of every 15 days, the length and weight of each fish from the tank were recorded using a meter rule and portable weighing scale. For SGR, the weight of each fish was measured at each sampling period. The various formulae used for measuring the above parameters are described by Kaviraj et al. (2004) and Saha et al. (2021) as follows:

Condition Factor (K, g/cm^3^) = (W/L^3^) × 100 [W = Body weight of fish (g), L = Body length of fish (cm)].

Hepatosomatic Index (HSI) = [Liver weight of fish (g) / Body weight of fish (g)] × 100.

Maturity Index (MI) = [Gonad weight of fish (g) / Body weight of fish (g)] × 100.

Specific Growth Rate (SGR, %/day) = [(Natural logarithm of final body weight of fish − Natural logarithm of initial body weight of fish) / Time interval] × 100.

### Analyses on total serum protein (TSP), total serum glucose (TSG) and hormones

The total serum protein (TSP) was measured following the method of Lowry et al. (1951). The total serum glucose (TSG) was estimated using One Touch Ultra Easy Glucometer (Johnson and Johnson). The analysis of testosterone (T) and 17β-estradiol (E_2_) was carried out using highly sensitive commercial ELISA kits (MyBiosource, Inc. San Diego, CA, USA; testosterone-MBS9424420 and 17β-estradiol-MBS1601666) following the company’s protocol. Growth hormone (GH) assay was done using the quantitative sandwich ELISA kit (MyBiosource, Inc. San Diego, CA, USA, MBS044656).

### Calculation of IBR

The Integrated Biomarker Response was calculated using the methodology described by Beliaeff and Burgeot (2002). IBR index was calculated using all the above-described parameters except MI, T and 17β-E. The non-inclusion of these parameters is related to the fact that both male and female data would benefit or penalise twice the same parameter. The full details can be found in the Supplementary Data.

### Statistical analyses

The statistical package SigmaPlot v12.5 was employed for data analysis. The differences in the test concentrations and control were subjected to one-way ANOVA followed by Dunnett’s Comparison Test to determine significant differences among the means (*p* < 0.05, Gomez and Gomez 1984). When possible, data transformation was used to achieve normality. When data did not show a normal distribution or homoscedasticity, the non-parametric test Kruskal–Wallis One Way Analysis of Variance on Ranks was used.

Data values higher or lower than the mean value, plus or minus two times the standard deviation, were considered outliers and withdrawn from analysis (Rousseeuw and Croux 1993). A two-way analysis of variance (two-way ANOVA) was performed to check for interactions between time and concentration.

## Results

### Haematological profiles and indices

**Fig. 1:**
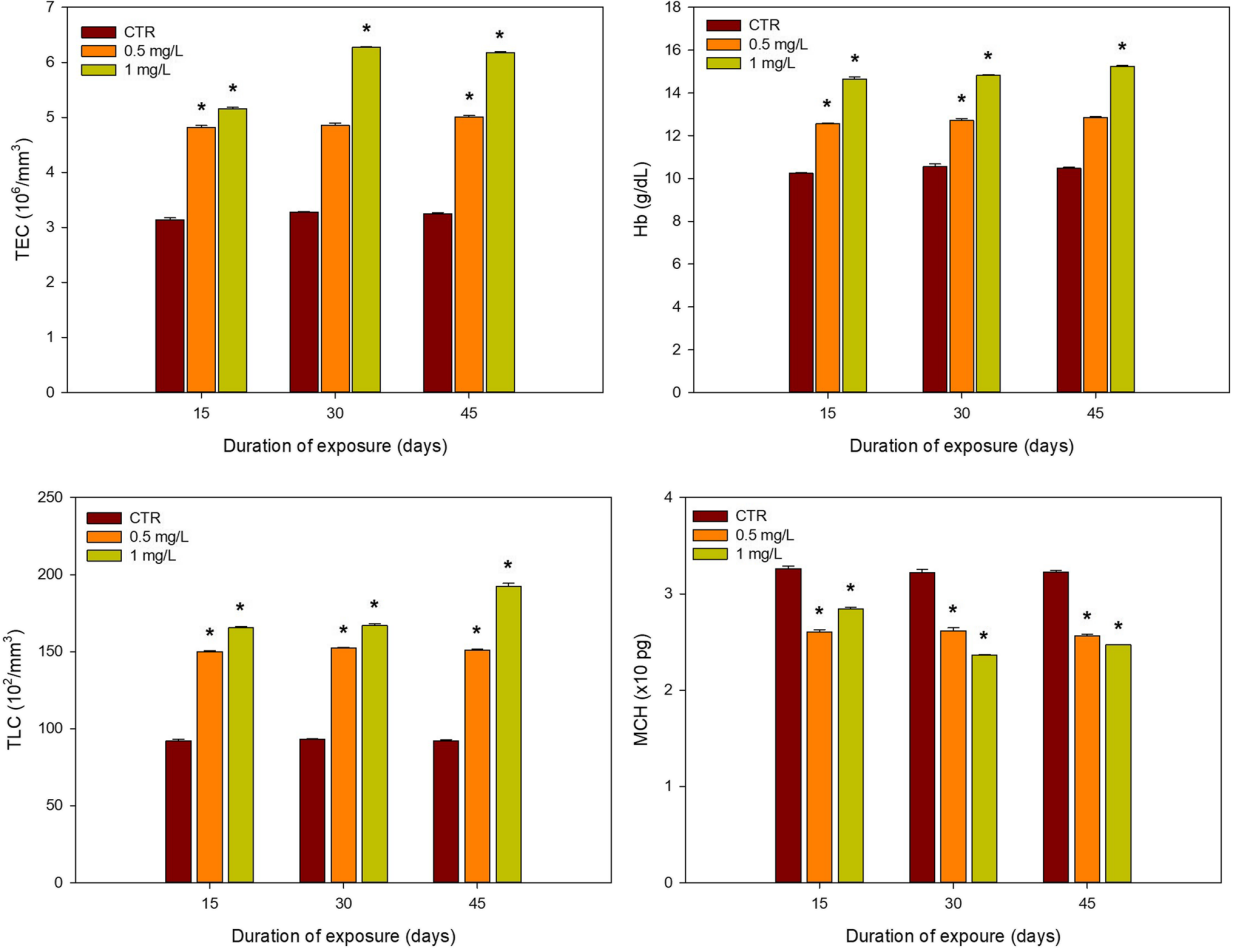
Mean and SD values (*n* = 4) of haematological profiles and indices of *Clarias batrachus* exposed to different concentrations of 2,4,6-TCP (0.5 and 1.0 mg/L) for different exposure times (15, 30 and 45 days). *denotes significant differences to control within the same exposure time (*p* < 0.05); TEC, total erythrocyte count; Hb, haemoglobin; TLC, total leucocyte count; MCH, mean corpuscular haemoglobin

The changes in different haematological parameters on chronic exposure to 2,4,6-TCP are presented in Fig. 1. A gradual increase pattern was observed for TEC, Hb and TLC for the two 2,4,6-TCP concentrations compared to the control (CTR), in opposition to MCH, where a decreasing pattern was observed. For TEC, a significant increase was observed for both concentrations after 15 and 45 days of exposure, whereas for 30 days, a significant increase was observed only for the higher concentration (1.0 mg/L). For Hb, a significant increase was observed for both concentrations after 15 and 30 days of exposure and for the higher concentration (1.0 mg/L) only after 45 days of exposure. For TLC, when compared to the control, significant increases were observed for both concentrations in all exposure times. On the other hand, when compared to the control, significant decreases for all concentrations and exposure times were observed for MCH. Due to the lack of homoscedasticity or normality, a two-way ANOVA could only be performed for TEC and MCH. On both parameters, a significant interaction was observed between time and concentration.

### Growth, reproductive endpoints and associated hormone assays

**Fig. 2:**
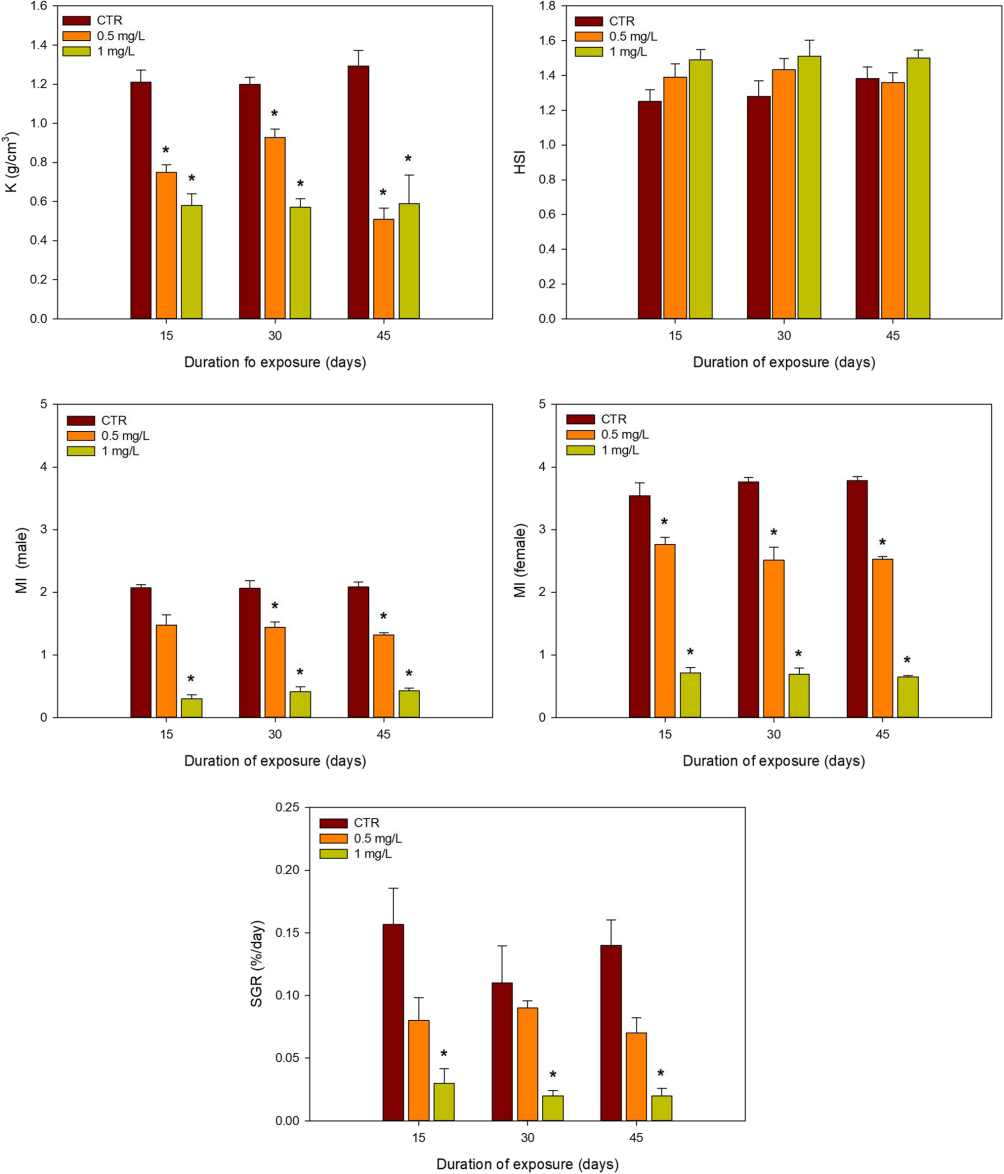
Mean and SD values (*n* = 4) of growth and reproductive endpoints of male and female *Clarias batrachus* exposed to different concentrations of 2,4,6-TCP (0.5 and 1.0 mg/L) for different exposure times (15, 30 and 45 days). *denotes significant differences to control within the same exposure time (*p* < 0.05); K, condition factor; HSI, hepatosomatic index; MI, maturity index; SGR, specific growth rate

The changes associated with the various growth and reproductive endpoints are summarised in Fig. 2.

The measured parameters (K, MI and SGR) showed a decreasing pattern. As for HSI, no differences were observed when treatments were compared to the control. For K, a significant decrease was observed for both concentrations compared to control during all the exposure time. No significant differences to control were found in HSI for both concentrations at different exposure times. A significant decrease in MI was observed for both male and female fish between the control and treatments for all the exposure periods, except for the lower concentration (0.5 mg/L) in male fish after 15 days of exposure. As for SGR, significant decreases to control were observed only for the highest concentration (1.0 mg/L) in all the exposure periods. The two-way ANOVA could only be performed for HSI, showing no significant interactions between time and concentrations.

**Fig. 3:**
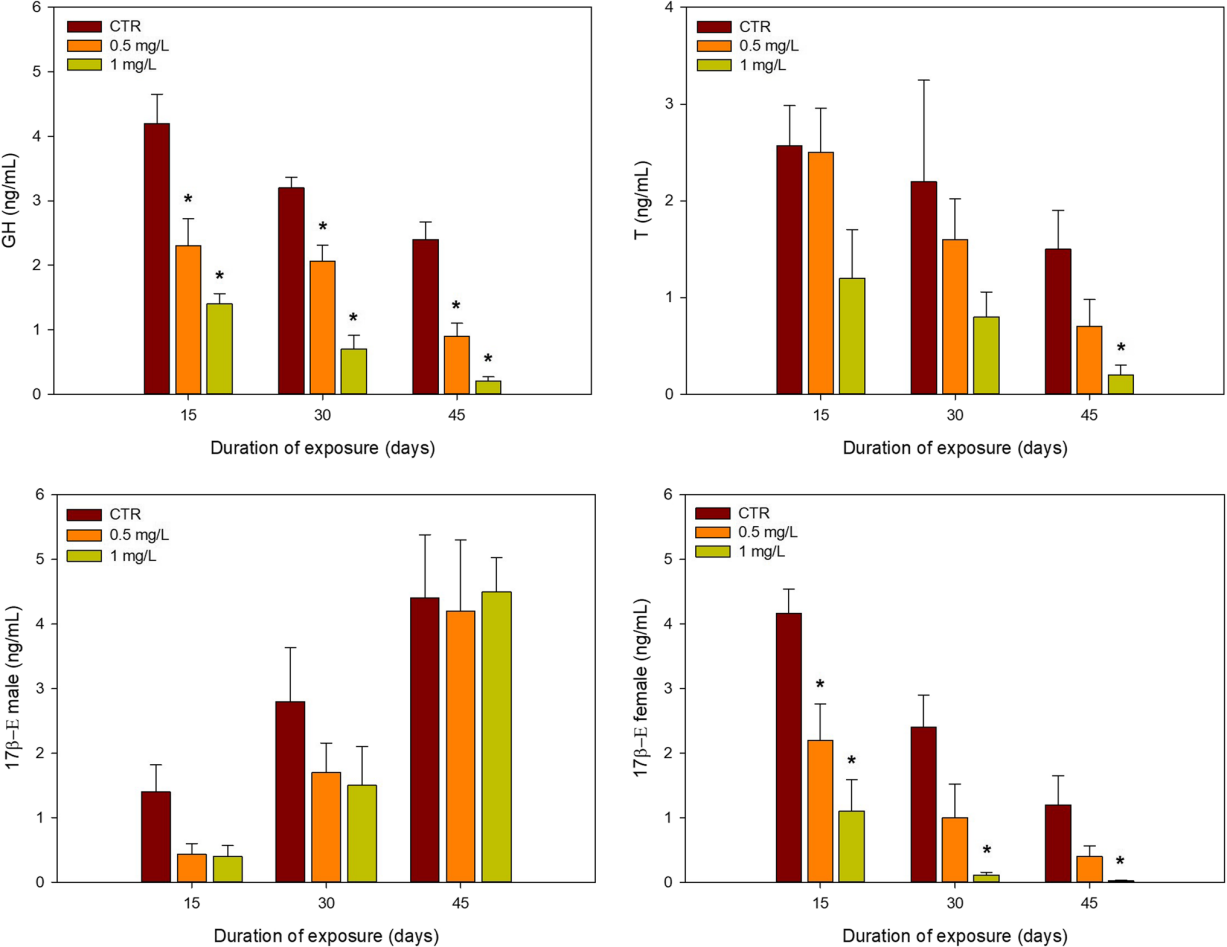
Mean and SD values (*n* = 4) of different hormones of *Clarias batrachus* exposed to different concentrations of 2,4,6-TCP (0.5 and 1.0 mg/L) for different exposure times (15, 30 and 45 days). *denotes significant differences to control within the same exposure time (*p* < 0.05); GH, growth hormone; T, testosterone; 17β-E, 17β-Estradiol

The sublethal effects of 2,4,6-TCP on GH, T and 17β-E for male and female in *C. batrachus* serum are presented in Fig. 3. For GH, a significant decrease was observed for both concentrations compared to control at all the exposure periods. For 17β-E in males, no significant differences were observed for both concentrations in all the sampling periods compared to the control. However, when reporting to 17β-E in females, a significant decrease was observed for the higher treatment (1.0 mg/L) at all sampling periods. As for the lowest concentration (0.5 mg/L) a significant decrease was observed only on the first 15 days of exposure. A two-way ANOVA could only be performed for 17β-E (males) and did not show any significant interaction between time and concentration.

### Total serum protein (TSP) and total serum glucose (TSG)

**Fig. 4:**
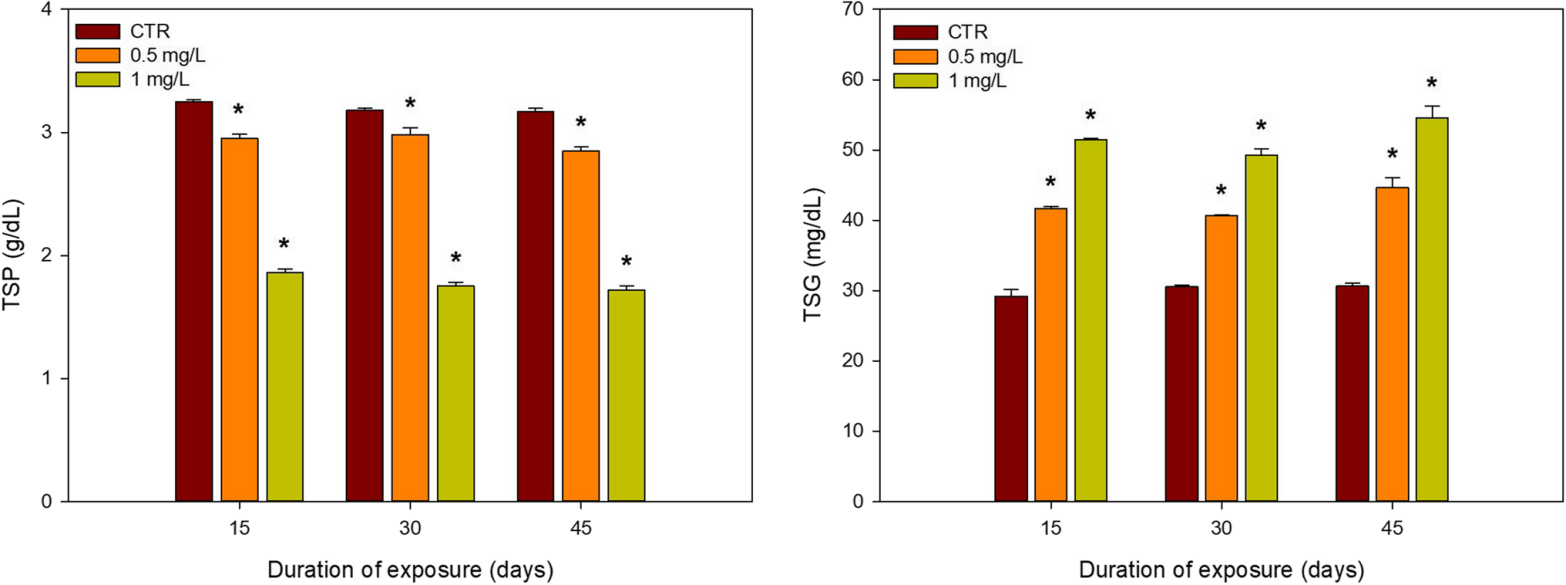
Mean and SD values (*n* = 4) of total serum protein (TSP) and total serum glucose (TSG) of male and female *Clarias batrachus* exposed to different concentrations of 2,4,6-TCP (0.5 and 1.0 mg/L) for different exposure times (15, 30 and 45 days). *denotes significant differences to control within the same exposure time (*p* < 0.05)

The changes associated with TSP and TSG levels are summarised in Fig. 4.

For TSP, a significant decrease to the control was observed for all the treatments during all the exposure periods. On the other hand, in the case of TSG, significant increases to the control were noted for both the treatments during all the sampling times. A two-way ANOVA could only be performed for TSP, showing no interaction between time and concentration.

### Integrated biomarkers response (IBR)

**Fig. 5:**
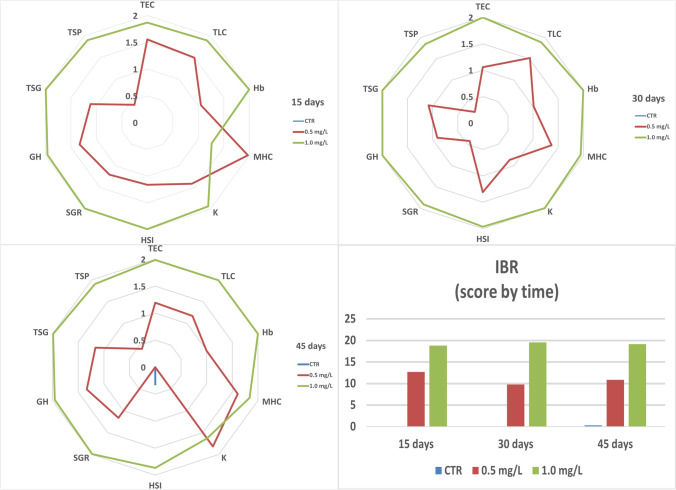
Integrated biomarker response by biomarker and time *of Clarias batrachus* exposed to different concentrations of 2,4,6-TCP (0.5 and 1.0 mg/L) for different exposure times (15, 30 and 45 days). TEC, total erythrocyte count; TLC, total leucocyte count; Hb, haemoglobin; MCH, mean corpuscular haemoglobin; K, condition factor; HSI, hepatosomatic index; SGR, specific growth rate; GH, growth hormone; TSG, total serum glucose; TSP, total serum protein

In Fig. 5 is presented the star plots for the IBR analyses. When looking to the different analysed parameters at the different sampling times, it is possible to observe an increasing pattern in the IBR score with the increase of 2,4,6-TCP concentrations. This increase is also reflected in the IBR scores over time. It is noteworthy that control scores are always zero or very close to it, whereas the treatments are much higher. This is even more evident for the IBR time scores, where the lower (0.5 mg/L) and higher (1 mg/L) concentration is 10 × and 20 × higher, respectively, than the control. The only score where the control had worse scores than the 2,4,6-TCP treatments was for the HSI parameter after 45 days of exposure.

## Discussion

The present research highlights the urgent need to address the uncontrolled generation of 2,4,6-TCP by various industries and its release and further accumulation in the aquatic ecosystems since it severely impacts freshwater organisms. The toxicity of 2,4,6-TCP acts as a potential hazard to the overall fish’s health by influencing haematological, growth, reproductive and biochemical endpoints, and associated hormones. This toxicity will eventually lead to an overall impact on the fish’s population.

### Haematological profiles and indices

The use of haematological endpoints is an important biomarker that can show the impact on fish by various toxic substances (Seibel et al. 2021). The exposure of *C. batrachus* to sublethal concentrations of 2,4,6-TCP changed the haematological profiles and indices of fish as showed by an increase in Hb, TEC and TLC and a decrease in MCH values. The observed increase in TEC values can be related to the increase of erythropoietin synthesis, related to the high demand for O_2_ transport, thus resulting in an enhanced metabolic rate (Modra et al. 2020). Similar studies on the effect of ten common pesticides on the African catfish, *Clarias gariepinus*, point out that the structural impairment of erythrocyte membrane leading to haemolysis drives the production of replacement erythrocytes to reduce the risk of anaemia (Amaeze et al. 2020). The spike in TEC may also be attributed to blood cell reserve coupled with cell shrinkage due to osmotic changes of blood caused by xenobiotics (Singh and Srivastava 2010). Another possible explanation for the observed results may be the damage to the gill membranes. This damage will impact gas exchange that will be reflected in TEC values (Hedayati and Tarkhani 2014).

Hb is another important endpoint since Hb is responsible for the oxygen-carrying capacity of blood (Rummer and Brauner 2015). The oxidative stress resulting from the exposure to contaminants may result in the formation of oxidised denatured Hb. As a result, the rise of Hb content may reflect the fish’s compensatory mechanism to replace the denatured Hb (de Moraes et al. 2018). The increase in Hb in the present study may also be related to the release of additional erythrocytes into the bloodstream to compensate for the oxygen saturation in the blood (Modra et al. 2020; Senthil Kumaran et al. 2011). Hypoxia may pose another explanation for the observed results, as it stimulates oxygen transport, increasing Hb content. Our results align with the works of Pimpão et al. (2007) and de Moraes et al. (2018), where an increase in Hb was also observed in the Brazilian fish, *Ancistrus multispinis*, and the neotropical fish, *Brycon amazonicus*, when exposed to deltamethrin and cypermethrin, respectively. When challenged by chemicals, an increase in nonspecific immunity is a primary response to maintain internal homeostasis (Netea et al. 2020).

TLC plays a significant role in the body’s defence against invasion by foreign bodies and infections and in producing, transporting and distributing antibodies involved in immune response (Ajima et al. 2015). The rise in TLC when exposed to 2,4,6-TCP may be a reflection of this phenomenon. A similar trend in TLC of the fish species *Oncorhynchus mykiss* exposed to phenol has also been reported by Louei Monfared and Salati (2013), supporting our results.

MCH indicates the content of Hb per red blood corpuscle (Javed et al. 2016). A decrease in MCH of *C. batrachus* exposed to 2,4,6-TCP observed in our study is corroborated by similar findings of Nte et al. (2011) in *Sarotherodon melanotheron* where a higher percentage of immature replacement erythrocytes in circulation has resulted in the development of hypochromic microcytic anaemia on exposure to industrial effluents. The obtained results in this study are also supported by the study of Oluah et al. (2020), where the African catfish, *Clarias gariepinus*, was exposed to the herbicide Ronstar.

### Growth, reproductive endpoints and associated hormone assays

Aquatic organisms are prone to growth inhibition on exposure to pulp and paper mill effluents (Hansson et al. 2014). The condition factor is a vital indicator for assessing fish’s health regarding internal energy reserve and food availability (Gandar et al. 2016). It also helps determine the toxic impact of various contaminants (Gandar et al. 2016). Hepatosomatic index (HSI) is considered a sensitive indicator of fish’s health status compared to the condition factor as it manifests changes at the tissue level (Gonino et al. 2019). Maturity index (MI) is also a common biomarker used to examine the deleterious effects of chemicals on gonadal tissues (Naderi et al. 2014). In the present study, the sublethal exposure of *C. batrachus* to 2,4,6-TCP resulted in an overall increase of HSI and a decrease of K, SGR and MI (male and female). The observed decrease in the K may be related to the exhaustion consumption of energy reserves of the muscle and simultaneous reduction in fat stored to meet up the energetic cost of homeostasis (Jamwal et al. 2018). Also, the fish’s energy requirement may increase to cope with detoxification processes (Osswald et al. 2013). Another mechanism behind the decrease in condition factor may involve damage to the olfactory system, thus resulting in disturbed feeding, change in metabolism, and energy allocation to maintain the growth of the fish species (Besson et al. 2020; Tierney et al. 2010). Chlorophenolic compounds, particularly pentachlorophenol, have been shown to cause toxic effects on the nervous system (Cheng et al. 2015). Additionally, impacts in K have been observed for several other stressors, for example, as presented in the study of Jamwal et al. (2018) that observed a similar decrease in fish condition factor exposed to dietary cadmium.

The liver is an important seat of metabolic activity in living organisms. The enhanced estrogenic activity in fish resulting in increased vitellogenin (VTG) production in the liver is a primary effect caused by the endocrine disrupting chemicals (EDCs, Tran et al. 2019). As the chlorophenolic compounds are a group of EDCs, it is expected that 2,4,6-TCP will increase VTG production in the liver. Although this study has not determined VTG levels, further research with 2,4,6-TCP should also consider this biomarker. This increase in VTG may be also associated to a rise in HSI (Zahran et al. 2020). A similar increasing trend in HSI was observed for other species, like *Oreochromis niloticus* and *Clarias gariepinus* exposed to paraquat and raw sewage, respectively (Figueiredo-Fernandes et al. 2006; Mdegela et al. 2010), or the species *Oryzias latipes* and *Takifugu fasciatus* exposed to octocrylene and copper, respectively (Wang et al. 2021; Yan et al. 2020). In their experiments, hepatosomatic index of males of *O. niloticus* exposed to paraquat was significantly higher at higher temperatures. On the other hand, the liver somatic index of *C. gariepinus* exposed to wastewater from sewage ponds exhibited a 2–threefold rise compared to the reference site (Figueiredo-Fernandes et al. 2006; Mdegela et al. 2010).

In our study, the reduction of MI of both sexes of *C. batrachus* subjected to chlorophenolic compounds may reflect the reduction of the gonad mass. Although a direct comparison to the MI values cannot be performed, it is still possible to compare the decreased percentage of values between control and treatments. Here, it is possible to observe similar decreases for both sexes. For the lowest exposure concentration (0.5 mg/L), the decrease was approximately 30%, whereas for the highest concentration (1.0 mg/L), it was approximately 85%, highlighting a similar impact in both sexes. The reallocation of energy reserves from gonad to other tissues or even the detoxification processes may have decreased the relative gonad size (Amat-Trigo et al. 2021; Sun et al. 2011). The decrease in male’s MI exposed to sublethal concentration of 2,4,6-TCP was probably due to a fall in testicular cell proliferation and spermatogenesis inhibition (Dong et al. 2018). As for females, ovarian maturation probably got disrupted due to decreased follicle and egg number indicative of suppressing oogenesis (Ji et al. 2013; Zhang et al. 2007). A similar MI trend was observed in *Cyprinus carpio* when subjected to 4-tert-butylphenol and dichlorvos, respectively (Barse et al. 2006; Mir et al. 2013), and for *Danio reriro* when exposed to tributyltin (Xiao et al. 2018).

Growth is an important index to measure the impact of various environmental stressors on fish (Poletto et al. 2018). In the present study, the sublethal exposure to 2,4,6-TCP significantly decreased the specific growth rate. For example, channel catfish (*Ictalurus punctatus*) and Nile tilapia (*Oreochromis niloticus*) exposed to different stressors like hypoxic conditions and elevated cadmium concentration exhibited reduced food intake that can be associated with a lower growth (Abdel-Tawwab and Wafeek 2010). The combination of a lower food intake and assimilation efficiency in the presence of stressor 2,4,6-TCP may even lead to anorexia. These findings are similar for other contaminants (i.e. sodium fluoride) as reported by Camargo (2003) and Kumar et al. (2007), or for *O. niloticus* exposed to nonylphenol as reported by Ismail and Mahboub (2016). The catabolism of existing biomolecules meets up the energy requirement and metabolic demand in stressed organisms (Abdel-Tawwab and Wafeek, 2010). This is consistent with our study that shows a significant decrease of TSP in *C. batrachus* exposed to 2,4,6-TCP after 45 days, as discussed below.

Hormones regulate the coordination between food consumption and somatic growth (Bertucci et al. 2019). GH and steroids play a crucial role in controlling the rate of voracity and fish growth (Canosa and Bertucci 2020; Kim et al. 2018). Insulin-like growth factor-I (IGF-I) stimulates growth on receiving a signal from GH, and their levels in the blood of fish are positively correlated (Medeiros et al. 2020). The present findings indicate that the circulating levels of GH, 17β-E and T in *C. batrachus* remarkably decreased with the increase in concentrations of 2,4,6-TCP after 45 days. Testosterone is a crucial hormone in all life stages, and as a result, any observed changes in their levels can show possible negative impacts on an organism’s health (Lewis et al. 2015). It is the main sex hormone for males responsible for growth, muscle mass, bone density and even cognition, whereas in females, its role is also related to cognition and bone metabolism, but mainly for ovarian and sexual function (Lewis et al. 2015). As for 17β-E, its hormonal role in females is related to the development of oocytes (Khara et al. 2014). 17β-E in male cannot be so strongly associated with reproduction as it is in females. Still, this hormone also interacts with adrenal glands, liver and fat, thus showing a possible impact in pathways not analysed in this study. The hypothalamus-pituitary–gonadal axis (HPG) regulates growth hormone and sex steroids (Acevedo-Rodriguez et al. 2018; Castañeda Cortés et al. 2014). 2,4,6-TCP might likely have inhibited GH secretion by acting at the level of hypothalamo-hypophysial axis. Moreover, brain amines also exert a control on the release of GH (Canosa et al. 2007), and toxicants affect the secretion of monoamine oxidase (Bridges et al. 2017). Thus, 2,4,6-TCP probably acts at the HPG level to inhibit GH secretion. 2,4-dichlorophenol and pentachlorophenol have been shown to lead to changes in 17β-E levels of zebrafish and T concentration in the serum of carps (Ma et al. 2012; Zhang et al. 2007) . Ma et al. (2012) reported that chlorophenols tend to disrupt the HPG axis affecting steroidogenesis and associated gene expression. Additionally, the significant decline in GH, T, 17β-E in 2,4,6-TCP exposed catfish is in accordance with the K, MI and SGR data.

Proteins are the body’s major biomolecules, with the liver playing a significant role in its metabolism. In our study, 2,4,6-TCP probably suppress TSP production in fish through the inhibition of protein synthesis and by preventing incorporation of amino acids in the polypeptide chain (Narra et al. 2017). Our finding is in accordance with the increased energy demand caused by chlorpyrifos and monocrotophos in *C. batrachus*, which resulted in a similar protein depletion pattern (Narra et al. 2017). Borges et al. (2007) and Öner et al. (2008) reported a similar decrease in serum protein of fish *Rhamdia quelen* and *Oreochromis niloticus* exposed to cypermethrin and copper, respectively. The rise in fish TSG levels may also be a secondary response in fish exposed to environmental stress (Narra et al. 2017). The increase in TSG levels observed in our study reflects a disturbance in carbohydrate metabolism due to the stimulation of the liver’s glucose 6-phosphatase activity. The rapid breakdown of liver glycogen or conversion of extrahepatic tissue proteins and amino acids to glucose has been previously reported (Narra 2016; Qian et al. 2019). Along with this liver’s activity, the increase in adrenocorticotrophic and glucagon hormones, coupled with decreased insulin levels, may also contribute to liver glycogen’s enhanced breakdown (Alkaladi et al. 2020). Chronic exposure to distillery effluent was found to result in increased blood sugar levels in *C. carpio* (Ramakritinan et al. 2005) and petroleum effluent in *Cirrhinus cirhossus* (Hamidi et al. 2021).

As presented before, the use of the IBR index in our study can help integrate all of the parameters into the toxicological impact that these organisms are dealing with, thus showing changes that could not be so easily noticed (Beliaeff and Burgeot 2002, Ferreira et al. 2015a, 2015b; Morgado et al. 2013). In our study, the treatments always showed worse scores than the control, and it was possible to observe a relation between the increase of 2,4,6-TCP’s concentration and the increase of scores. As for what reports to the IBR scores within the different sampling times, it is possible to observe the same pattern exposure to increasing 2,4,6-TCP’s concentrations leading to worse scores (10 × and 20 × higher than the control for 0.5 and 1.0 mg/L). It is also noteworthy to highlight that each given parameter may display certain scores that may not show control as always the best score. This is the case of HSI for the control after 45 days of exposure and can be seen in many other studies (e.g. Ferreira et al. 2015a; Morgado et al. 2013). Despite the lack of IBR data for the impact of chlorophenols on fish, previous studies have already proven that IBR can be a useful tool to assess laboratory work (Madeira et al. 2016; Morgado et al. 2013).

## Conclusions

The present research shows the effects of 2,4,6-TCP on the fish species *Clarias batrachus*. 2,4,6-TCP exposure resulted in altered haematological, biochemical, growth and reproductive parameters using the IBR index to integrate all of the toxicological effects. This is the first study showing its effects on higher organisational levels, thus underlying the mechanism behind the changes in haematological, biochemical, growth and reproductive indices. Without exceptions, the results showed that 2,4,6-TCP had a deleterious impact in all of the tested parameters in the catfish *C. batrachus*. This impact occurred even at the lowest concentration (0.5 mg/L) for the shortest exposure time (15 days). These findings were further highlighted by the IBR index, which showed up to 20 × worse scores than the control for all treatments and sampling times. The present data can then be used for regulatory policymakers to regulate better the industry and their illegal discharge of effluents that may be rich in 2,4,6-TCP. This becomes even more important for local populations that directly use this catfish species as food and medicinal sources. They may see their population numbers decrease or inadvertently ingest this type of pollutants. Nonetheless, future research is still needed, for example, on the fine structure of selected fish tissues to corroborate the present findings of 2,4,6-TCP toxicity and adopt specific biological methods to mitigate 2,4,6-TCP toxicity.

## Supplementary Information

Below is the link to the electronic supplementary material.

Supplementary file1 (DOCX 21 KB)

## Data Availability

The datasets used and/or analysed during the current study are available from the corresponding author on reasonable request.
