# Supplementary material for: Effects of 2,4,6-Trichlorophenol on Clarias batrachus: a biomarkers approach
Source: Environ Sci Pollut Res Int. 2022 Feb 17;29(31):47011–24. doi: 10.1007/s11356-022-19213-y (PMC9232441; doi:10.1007/s11356-022-19213-y)
Supplement: Supplementary file 1 — Supplementary file1 (DOCX 21 KB) [file 11356_2022_19213_MOESM1_ESM.docx]

Effects of 2,4,6-Trichlorophenol on *Clarias batrachus*: a biomarkers approach.

**Dip Mukherjee^1*^, Nuno G.C. Ferreira^2,3*^, Nimai Chandra Saha^4^**

^1^ Department of Zoology, S.B.S. Government College, Hili, Mera Aptair, Balurghat, Dakshin Dinajpur-733126, West Bengal, India.

^2^ Cardiff University, School of Biosciences, Cardiff, CF10 3AX, United Kingdom.

^3^ CIIMAR - Terminal de Cruzeiros de Leixões, Av. General Norton de Matos S/N - 4450-208 Matosinhos, Portugal

^4^ The University of Burdwan, Fishery and Ecotoxicology Research Laboratory, Vice Chancellor’s Research Group, Department of Zoology, University of Burdwan, Purba Barddhaman, West Bengal, India.

*- corresponding authors: [dip.m@rediffmail.com](mailto:dip.m@rediffmail.com); [Ferreiran@cardiff.ac.uk](mailto:Ferreiran@cardiff.ac.uk)

SUPPLEMENTARY DATA

**Table S1:** Physico-chemical parameters of the water used in different treatments (CTR, 0.5 mg/ L and 1.0 mg/ L of 2,4,6-TCP) during the chronic toxicity study. (mean±SD are values of four replicates)

| **Exposure Period** | **15days**  **(mean± SD)** | | | **30days**  **(mean±SD)** | | | **45days**  **(mean±SD)** | | |
| --- | --- | --- | --- | --- | --- | --- | --- | --- | --- |
| **Concentration**  **(mg/L)** | **CTR** | **0.5** | **1.0** | **CTR** | **0.5** | **1.0** | **CTR** | **0.5** | **1.0** |
| **Temperature (°C)** | 26.3  ± 0.1 | 26.2  ± 0.1 | 26.0  ± 0.2 | 26.3  ± 0.1 | 26.2  ± 0.1 | 26.2  ± 0.1 | 26.3  ± 0.2 | 26.2  ± 0.1 | 26.2  ± 0.1 |
| **pH** | 7.2  ± 0.1 | 8.0  ± 0.2 | 8.3  ± 0.1 | 7.1  ± 0.1 | 8.0  ± 0.2 | 8.3  ± 0.1 | 7.2  ± 0.1 | 8.1  ± 0.5 | 8.3  ± 0.1 |
| **Dissolved Oxygen (mg/L)** | 6.0  ± 0.5 | 5.2  ± 0.2 | 5.4  ± 0.1 | 6.1  ± 0.1 | 5.4  ± 0.1 | 5.2  ± 0.1 | 5.4  ± 0.3 | 5.4  ± 0.2 | 5.4  ± 0.2 |
| **Hardness**  **(mg/L)** | 115  ± 2.5 | 136  ± 2.2 | 137  ± 4.0 | 116  ± 2.6 | 131  ± 2.7 | 137  ± 1.8 | 118  ± 2.4 | 131  ± 2.2 | 140  ± 1.3 |
| **Alkalinity (mg/L)** | 152  ± 2.1 | 209  ± 6.7 | 212  ± 4.2 | 148  ± 3.1 | 206  ± 4.2 | 205  ± 4.1 | 150  ± 3.4 | 205  ± 1.0 | 206  ± 4.4 |

## Integrated Biomarker Response (IBR)

The integrated biomarker response (IBR) was calculated to integrate all parameters into a global/general index, according to Beliaeff and Burgeot (2002). The IBR is calculated by summing up triangular Star Plot areas calculated for every two-neighbouring data.

General mean (*m*) and the standard deviation (*s*) of all data regarding a given parameter was calculated, followed by standardisation to obtain *Y*, where *Y* = (*X* - *m*)/*s*, and *X* is the mean value for the biomarker at a given concentration. Then *Z* was calculated using *Z* = -*Y* or *Z* = *Y*, in the case of a biological effect corresponding respectively to inhibition or stimulation. The parameters TEC, TLC, Hb, HSI and TSG were assumed to increase upon the exposure to 2,4,6-TCP. As for MCH, K, SGR and GH assumed to decrease with the exposure to the stressor.

The score (*S*) was calculated by *S* = *Z*+|*Min*|, where *S* ≥0 and |*Min*| is the absolute value for the minimum value for all calculated *Y* in a given biomarker at all measurements made.

Star plots were then used to display Score results (*S*) and to calculate the integrated biomarker response (IBR) as:

$$IBR= \sum_{i=1}^{n} A_{i}$$

$$A_{i}= \frac{S_{i}}{2} \sin\beta\left( S_{i}\cos\beta+ S_{i+1}\sin\beta\right)$$

$$\beta= \tan^{-1} \left( \frac{S_{i+1}\sin\alpha}{S_{i}- S_{i+1}\cos\alpha} \right)$$

where *S_i_* and *S_i+1_* are two consecutive clockwise scores (radius coordinates) of a given star plot; *A_i_* corresponds to the area the connecting two scores; *n* the number of biomarkers and energy reserves used for calculations; and α = 2п/*n*. The IBR index was calculated using the parameters always in the same order for all sampling times.
